# Supplementary material for: The Impact of Neurological Complications in Endocarditis: A Systematic Review and Meta-Analysis
Source: J Clin Med. 2024 Nov 22;13(23):7053. doi: 10.3390/jcm13237053 (PMC11642100; doi:10.3390/jcm13237053)

Supplemental material

Table S1: MINORS criteria for the selected studies included in the meta-analysis

References	Q1	Q2	Q3	Q4	Q5	Q6	Q7	Q8	Q9	Q10	Q11	Q12	overall
Arregle *et al.* 2021 [12]	2	2	2	2	2	2	1	0	2	2	2	2	21
Cooper *et al.* 2009 [5]	2	2	2	2	2	2	0	0	2	2	2	2	20
Diab, *et al.* 2016 [13]	2	0	0	2	2	2	2	0	2	2	2	2	18
Fukuda, *et al.* 2014 [14]	2	0	0	2	2	0	0	0	2	2	2	2	14
Garcìa-Cabrera, *et al.* 2013 [15]	2	2	2	2	2	2	1	0	0	2	2	2	19
Grabowski, *et al.* 2010 [16]	2	0	2	2	2	2	2	0	2	2	2	1	19
Heiro, *et al.* 2007 [17]	2	0	0	2	2	2	0	0	2	2	2	2	16
Huang, *et al.* 2023 [18]	2	0	0	2	2	2	2	0	2	2	2	2	18
Kim, *et al.* 2017 [19]	2	0	0	2	2	2	0	0	2	2	2	2	16
Lee Su Jin, *et al.* 2014 [20]	2	0	0	2	2	2	2	0	2	2	2	2	18
Lee Seung-Jae *et al.* 2014 [21]	2	0	0	2	2	2	2	0	2	2	2	2	18
Misfeld, *et al.* 2014 [22]	2	0	0	2	2	2	0	0	2	2	2	2	16
Okazaki, *et al.* 2013 [23]	2	0	0	2	2	2	0	0	2	2	2	2	16
Pinto, *et al.* 2024 [24]	2	2	2	2	2	2	1	0	2	2	2	2	21
Roder, *et al.* 1997[25]	2	0	0	2	2	2	0	0	2	2	2	2	16
Ruttmann, *et al.* 2020 [26]	2	2	0	2	2	2	0	0	2	2	2	2	18
Santoshkumar *et al.* 1996 [27]	2	0	0	2	2	2	0	0	2	2	1	1	14
Scheggi, *et al* 2022[28]	2	0	0	2	2	2	0	0	2	2	2	2	16
Selton-Suty, *et al* 2016[29]	2	0	0	2	2	2	0	0	2	2	2	2	16
Sonneville, *et al* 2011[30]	2	2	2	2	2	2	2	0	2	2	2	2	22
Suzuki, *et al* 2017[31]	2	2	0	2	2	2	0	0	2	2	2	2	18
Thuny, *et al* 2007[32]	2	2	2	2	2	2	2	0	2	2	2	2	22
Tsai, *et al* 2024[33]	2	0	0	2	2	2	0	0	2	2	2	2	16
Wilbring *et al* 2014[34]	2	0	0	2	2	2	2	0	2	2	2	2	18
Zaballos *et al* 2024[35]	2	2	2	2	2	2	0	0	2	2	2	2	20

Q: question

Table S2: Subtypes of neurological complications and their relation to the primary outcome

References	NC subtypes according to study design	NC subtypes used for analysis (M: major; m:minor)	OR of the primary outcome
Diab et al, 2016 (13)	Major CVC (mRS >3)	M	0.85 (0.55-1.30)
Garcia-Cabrera et al, 2013 (15)	Neurological complications together		1.58 (1.23-2.02)
	Encephalistis/meningitis		0.94 (0.61-1.45)
	Small ischemic complications	m	1.41 (0.93-2.19)
	Moderate-severe ischemic complications	M	1.63 (1.19-2.22)
	Cerebral hemorrhage		1.73 (1.10-2.71)
Huang et al , 2023 (18)	Symptomatic NC	M	2.884 (2.574 – 3.213)
Lee Seung-Jae et al, 2014 (20)	Minor CVC	m	0.856 (0.253-2.894)
	Major CVC (initial mRS >=3)	M	2.865 (1.254-6.548)
Ruttmann E. et al, 2020 (26)	Uncomplicated stroke	m	1.18 (0.79–1.77)
	Complicated stroke	M	2.1 (1.24–3.54)
Selton-Suty et al, 2016 (29)	Asymptomatic NC	m	0.393 (0.120-1.281)
	Symptomatic NC	M	2.499 (1.623-3.894)
Sonneville et al, 2011 (30)	Symptomatic NC		1.21 (0.63–2.33)
	Neurological failure GCS <10	M	7.41 (2.89-18.96)
Suzuki et al, 2017 (31)	Complicated stroke	M	10.763 (1.232-155.872)
Thuny et al, 2007 (32)	Silent cerebral embolism or TIA	m	0.9 (0.46–1.61)
	Stroke	M	1.6 (1.02–2.65)

NC: neurological complications, CVC: cerebrovascular complications, mRS: modified Rankin Score, GCS: Glasgow coma scale, TIA: transient ischemic attack.

Table S3: Meta-regression analysis on the primary outcome

Moderator	Beta	P value for interaction
Male sex	-0.01	0.450
Mean age	-0.02	**0.003**
Prosthesis involvement	-0.00	0.854
Right heart valve involvement	0.02	0.645
Mitral valve involvement	0.01	0.124
Aortic valve involvement	-0.01	0.073
S.A positive emoculture	-0.00	0.675
Presence of valvular abscess or local complications	0.00	0.629
Valve surgery	-0.00	0.330
Early surgery (< 7 days)	-0.00	0.312
Heart failure at admission	0.00	0.937

SA: S.Aureus

Figure S1: Funnel plot

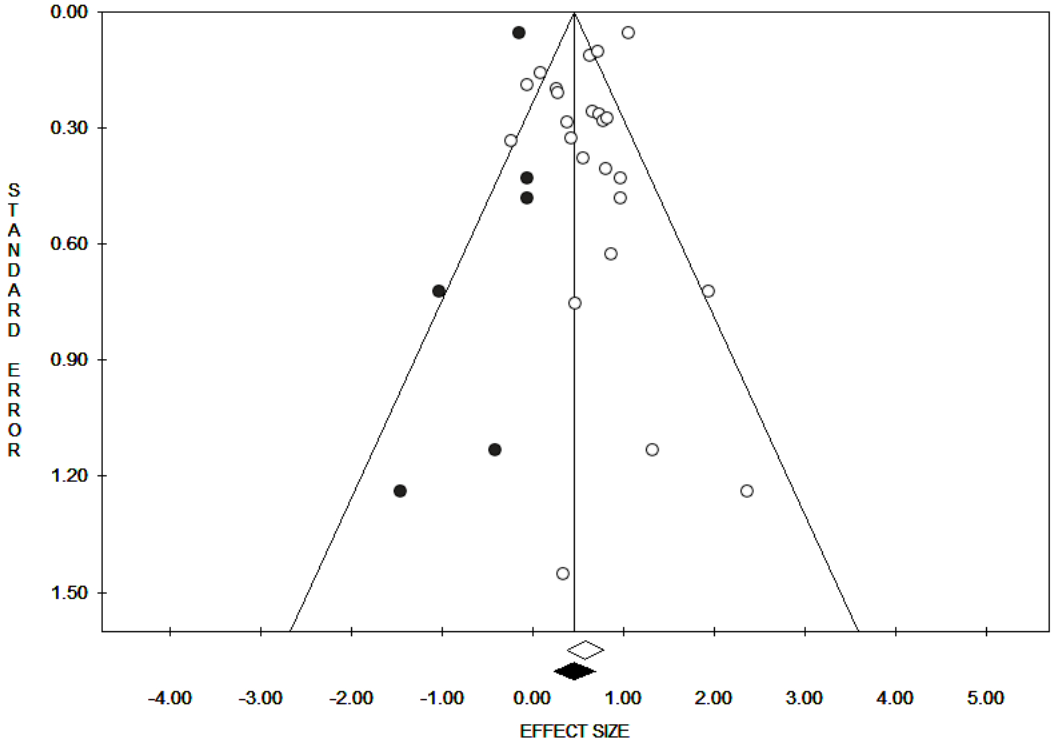

Supplement: Supplementary file 1 [file jcm-13-07053-s001.zip › jcm-3312469-supplementary.pdf]
